# The Impact of m6A RNA Modification in Therapy Resistance of Cancer: Implication in Chemotherapy, Radiotherapy, and Immunotherapy

**DOI:** 10.3389/fonc.2020.612337

**Published:** 2021-02-25

**Authors:** Omprakash Shriwas, Pallavi Mohapatra, Sibasish Mohanty, Rupesh Dash

**Affiliations:** ^1^ Institute of Life Sciences, Bhubaneswar, India; ^2^ Manipal Academy of Higher Education, Manipal, India; ^3^ Regional Center for Biotechnology, Faridabad, India

**Keywords:** m6A methylation, cisplatin, PD-1, METTL3, YTHDF1, ALKBH5

## Abstract

m6A RNA methylation, which serves as a critical regulator of transcript expression, has gathered tremendous scientific interest in recent years. From RNA processing to nuclear export, RNA translation to decay, m6A modification has been studied to affect various aspects of RNA metabolism, and it is now considered as one of the most abundant epitranscriptomic modification. RNA methyltransferases (writer), m6A-binding proteins (readers), and demethylases (erasers) proteins are frequently upregulated in several neoplasms, thereby regulating oncoprotein expression, augmenting tumor initiation, enhancing cancer cell proliferation, progression, and metastasis. Though the potential role of m6A methylation in growth and proliferation of cancer cells has been well documented, its potential role in development of therapy resistance in cancer is not clear. In this review, we focus on m6A-associated regulation, mechanisms, and functions in acquired chemoresistance, radioresistance, and resistance to immunotherapy in cancer.

## Introduction

Cancer remains a key public health concern posing a major threat to the world’s population. According to Siegel et al. each year approximately1,806,590 new cases of cancer are being diagnosed and around 606,520 people lose their life to cancer alone in the United States (1). The most frequently used therapeutic regimens for cancer include surgery, chemotherapy, radiotherapy, and more recently immunotherapy (2, 3). Although there have been breakthroughs and successes in treating specific types of cancer, most strategies have not proven as efficacious as hoped or predicted. One of the major causes of failure to treat cancer is a lack of understanding of the molecular mechanism behind the therapy resistance. Chemoresistance is one of the major factors for treatment failure in cancer. The chemotherapy drugs efficiently eradicates the rapidly dividing cells but poorly eliminates the slow dividing cells, particularly when lower dose of drug is provided to balance its cytotoxic effect on normal or non-transformed cells. This population of cells, which partially responds to chemotherapy drug, contributes to development of chemoresistance. Ultimately, the patients experience tumor relapse, which culminates with continued tumor growth and metastatic spread (4). The chemoresistance can be divided into two categories: “intrinsic chemoresistance” where cancer cells are inherently resistant prior to chemotherapy and “acquired chemoresistance” where cancer cells acquire resistance during prolonged treatment with agents that they initially displayed sensitivity. The chemoresistant phenotype of cancer cells can be attributed due to impaired apoptosis, altered cellular metabolism, decreased drug accumulation, reduced drug-target interactions, and increased populations of cancer stem cells (2). However, these are the endpoint events and the causative factors responsible for acquired chemoresistance is yet to be known. Similarly, cancer cells develop resistance against ionizing radiation (radioresistance) by enhancing DNA damage response, altering the expression of oncogene/tumor supressors, manipulating the tumor microenvironment, and by regulating the cell cycle (5). Understanding the molecular mechanisms behind the therapy resistance will enable us to overcome the drug resistance in cancer.

With the discovery of methylation of O6-methylguanine-DNA methyltransferase (MGMT) that sensitizes glioblastoma multiforme (GBM) cells to temozolomide, epigenetic alterations have been extensively studied to uncover the molecular mechanism behind therapy resistance (6, 7). Approximately 100 different types of modifications can be observed at RNA level, but m6A modification of RNA has gathered much attention. Since then, researchers pushed their focus and discovered Writer, Reader, and Erasers for RNA modification (8). Advancement of techniques like high throughput sequencing enabled the scientific community to uncover m6A enrichment at RNA. Modification of m6A in transcriptome is not random, but happens at a consensus sequences like DRACH (D = G, A, or U; R = G or A; H = A, C, or U), which are enriched mostly in CDS as well as 3’UTR region (9, 10). RNA methylation occurs on several sites including 5-methylcytosine (m5 C), 7-methylguanosine (m7 G), m1 G, m2 G, m6 G, N1 - methyl adenosine (m1 A), and m6 A (11). The m6A modification occurs *via* “writers” (i.e., m6A methyltransferases), recognized by “readers” (i.e., m6A-binding proteins), and removed by “erasers” (i.e., m6A demethylases) in eukaryotes (12). Methyltransferase-like 3 (METTL3), METTL14, Wilms tumor 1-associated protein (WTAP), KIAA1429, RNA-binding motif protein 15 (RBM15), and zinc finger CCCH domain-containing protein 13 (ZC3H13) forms the “writer” complex that initiates the m6A modification (13, 14). YT521-B homology(YTH) proteins, insulin-like growth factor 2 mRNA binding proteins (IGF2BPs), eukaryotic initiation factor 3 (eIF3), heterogeneous nuclear ribonucleoproteins (HNRNPs), and fragile X mental retardation proteins (FMRPs) are included under “reader” complex that recognizes the m6A RNA modification and initiates downstream signaling (13). Obesity-associated protein (FTO) and alkB homolog 5 (ALKBH5) stimulate the demethylation process and are included under “eraser” complex (15, 16). Extensive studies on m6A modification indicated toward its contribution in regulation of mRNA (17), long non-coding RNA (lncRNA) (18), microRNA (19), and circular RNA (circRNA) (20). m6A modification being an important RNA regulatory mechanism has been proved to play a critical role in regulating RNA processing, transportation, translation, and decay. Methyltransferase-like 3 (METTL3) methylates pri-miRNAs, enabling them to be recognized by RNA-binding protein DGCR8 and thereby leading to miRNA maturation (21). The global RNA modification study suggests that RNA demethylase FTO was found to regulate pre-mRNA processing including alternative splicing and 3′ UTR processing (22). Studies also revealed that m6A is added to exons in nascent pre-mRNA and its addition in the nascent transcript is a determinant of cytoplasmic mRNA stability (22). Interestingly, selective down regulation of METTL3 reduces the translation of mRNAs bearing 5’ UTR methylation. In this study, it was found that ABCF1 coordinates with METTL3 in m6A-facilitated and eIF4F independent mRNA translation (23), demonstrating the role of m6A methylation in mRNA translation. m6A-binding protein YTHDC1 mediates export of methylated mRNA from the nucleus to the cytoplasm, demonstrating the potential role of m6A modification in RNA translocation (24). There is emerging evidence indicating that m6A modification is strongly associated with acquired therapy resistance in cancer. In this review, we have focused on the mechanisms of RNA m6A modification-associated therapy resistance and possible approaches to overcome it.

## Implication in Chemoresistance

Reprogramming chemoresistant cells to undergo drug induced apoptosis is a viable approach to treat recurrent neoplastic diseases. This can be achieved by selective downregulation of anti-apoptotic factors or activation of pro-apoptotic factors in tumor cells (2). Among several novel approaches, modulation of N6-methyladenosine(m6A) RNA modification was found to be an important strategy in various types of cancer cells to overcome drug induced cell death. Various studies indicate that m6A modification confers drug resistance by regulating ABC transporters directly on transcript level or *via* upstream signaling pathways (19). Similarly, studies suggested that m6A modification affects the expression of BCL-2 with variable outcomes depending on the different cancer types (25, 26). Recent studies indicate that the m6A modification is involved in the maintenance of CSCs in tumors, leading to drug resistance and recurrence. Considering the potential role of m6A RNA modification in development of chemoresistance, it can be a viable therapeutic target to overcome chemoresistance.

### Cisplatin Resistance and m6A Modification

Cisplatin is the first line of treatment for several neoplasms. In 1965, Barnett Rosenberg accidently discovered the role of cisplatin in cell division. Further studies substantiated that it is the most promising agent for treatment of cancer (27). Writer protein METTL3 is involved in acquired cisplatin resistance by regulating TRIM11 expression. Methylated RNA immunoprecipitation (Me-RIP) study suggests that TRIM11 m6A level was higher in cisplatin resistant cells compared to sensitive cells in nasopharyngeal carcinoma (NPC) lines. Depletion of METTL3 results in reduced TRIM11 expression that sensitizes NPC lines to cisplatin (28). Similarly, METTL3 enhances the YAP1 m6A methylation at mRNA level and stabilize its expression in human lung cancer lines. The elevated YAP1 mediates cisplatin resistance in NSCLC (19). Reader protein YTHDF1 depletion mediates cisplatin resistance in NSLCC through KEAP1/NRF2/AKR1C1 axis and higher expression of YTHDF1 showed better clinical outcome of NSCLC patient (29). Erasers also play an important role in acquired cisplatin resistance in several neoplasms. FTO demethylates β-catenin mRNA and stabilizes the β-catenin in cervical squamous cell carcinoma, thereby inducing chemo-radio therapy resistance (30). In our study, we found that ALKBH5 is directly regulated by human RNA helicase DDX3, which leads to decreased m6A methylation in FOXM1 and NANOG nascent transcript that contribute to cisplatin resistance in OSCC (31).

### Kinase Inhibitor and m6A Modification

Kinase inhibitors have emerged as a potential strategy for treatment of cancer. Currently, several FDA approved kinase inhibitors are being evaluated in different phases of clinical trials to treat cancer (32). m6A RNA modifications play an important role in acquiring resistance against kinase inhibitors. A comparative study in NSCLC cell lines suggests that higher m6A enrichment scores can be found in afatinib resistant lines as compared with sensitive cells (33). Similarly, RNA methylation status was compared between TKI (tyrosine kinase inhibitor) resistant and sensitive cells and it was found that cells having hypomethylation showed greater tolerance for TKI and better growth rate. FTO- enhances mRNA stability of prosurvival transcripts and further induces resistance to tyrosine kinase inhibitors (TKIs) in leukaemia cells (26). Depletion of METTL3 induces sorafenib resistance in human liver cancer lines. Mechanistically, it was found that depletion of METTL3 reduces the stabilization of FOXO3 mRNA and ectopic overexpression of FOXO3 restores sorafenib sensitivity (34).

### 5-Fluorouracil Resistance and m6A Modification

5-Fluorouracil (5FU) is a widely used anticancer drug in many cancers. It is an analogue of uracil, which gets incorporated into nucleic acids and interfere with nucleotide metabolism (35, 36). For treatment of several neoplasms, the common chemotherapy regimen involves TPF (Taxol, Platinum, and Fluorouracil) or FOLFOX (Folinic acid, Fluorouracil and Oxaliplatin) (37). The role of m6A in 5 FU resistance is not well studied except few reports, which indicates m6A RNA modification augments the chemosensitivity of 5 FU. METTL3 knockdown increases the 5FU sensitivity in pancreatic ductal adeno carcinomas (38). Similarly, reader protein YTHDF1 knockdown results in enhanced 5FU sensitivity in colorectal cancer (39).

### PARP Inhibitor and m6A Modification

DNA damage is a common mode of action for most of the anticancer drugs and absence of an efficient DNA repair system in cancer cells leads to drug induced death. PARP (poly (ADP-ribose) polymerase) is a key enzyme that plays important roles in DNA damage response. PARP1 identifies and interacts with single stranded DNA damage through its DNA binding domain. Further, PARP1 synthesizes poly(ADP) ribose (PAR) and transfers it to acceptor proteins. PAR recruits repair proteins to the damaged DNA site. Henceforth, PARP1 has been established as an important target for cancer therapy. As many as 8 different PARP inhibitors are in different phases of clinical trial against various neoplasms (40–42). PARP inhibitors generally bind to the cofactor and catalytic domain and inhibits its enzyme activity (43). The most commonly used PARP1 inhibitors are Olaparib, Rucaparib, Niraparib, and Talazoparib (44). Olaparib is the first inhibitor used for clinical trial in BRCA 1 mutant solid tumor (45). Only few studies with m6A modification and PARP1i resistance are available in literature. Fukumoto et al. (2019) performed a global m6A modification profiling and found that in BRCA-mutated lines, m6A modification stabilizes the expression of FZD10 mRNA, which ultimately contributes to PARP inhibitor resistance. Mechanistically it was found that enhanced expression of *FZD10* leads to activation of Wnt/β-Catenin signalling. ALKBH5 and FTO knockdown decreased FZD10 mRNA stability and sensitize the cell to PARP inhibitor (46). This study clearly indicated that m6A modification plays a crucial role during the development of PARPi resistance.

### Gemcitabine and m6A Modification

Gemcitabine, a pyrimidine analogue, is used as chemotherapeutic regimen in several neoplasms including pancreatic, ovarian, breast, bladder, and small lung carcinoma. Moreover, Gemcitabine enhances the survival rate of pancreatic cancer patients up to 20% (47). Interestingly, Gemcitabine decreased the expression of ALKBH5 in PDAC xenografts. Ectopic overexpression of ALKBH5 sensitizes PDAC lines to Gemcitabine. On the other hand, knockdown of ALKBH5 in PDAC lines enhanced cell growth, proliferation, and migration. RNA immunoprecipitation followed by sequencing data suggests that in ALKBH5 knock down cells, increased m6A modification at the 3′ UTR region of the WIF-1 (Wnt inhibitory factor 1) mRNA can be observed. Henceforth, the expression of WIF-1 is down regulated in ALKBH5 KD cells, which in turns activate Wnt pathway and enhances the expression of Wnt target genes like C-MYC, Cyclin D1, and MMP-2 (48). On the contrary, knock down of METTL3 enhanced the sensitivity towards many chemotherapeutic drugs including gemcitabine (38).

## Implication in Immunotherapy

Interestingly, m6A RNA modification also plays an important role in regulating immune response in cancer patients. He et al. analyzed the RNA sequencing data of 24 different m6A regulators in 775 breast cancer patients from TCGA database and categorized them in two subgroups. One group had a lower RNA methylation status (RM1) and other had a high methylation status (RM2). The RM1 group showed shorter overall survival rate and higher enrichment of PI3K and KRAS signalling. On the other hand, the RM2 group showed higher numbers of tumor-infiltrating CD8+ T cells, helper T cells, and activated NK cells, but lower expressions of PD-L1, PD-L2, TIM3, and CCR4 than RM1 group (49). Similarly, the study by Winkler et al. suggested that m6A modifications serve as a negative regulator of interferon response by modulating the turnover of interferon mRNAs (50). Writer, reader, and erasers play important roles in immune surveillance. Rubio et al. suggest that writer METTL14 depletion induces IFNβ1 production, whereas ALKBH5 depletion reduces IFNβ1 production (51). T cell homeostasis is very important for any kind of defense balance, but depletion of writer METTL3 in CD4 cells hampered the homeostasis of T cells (52). METTL3 depletion in dendritic cells resulted in impaired maturation of dendritic cell and led to weak costimulatory signal by CD40-CD80 as well as exerted reduced T cell stimulation (53).The reader protein YTHDF1 regulates immune response in melanoma cancer. YTHDF1 deletion in mice showed slower growth of melanoma and higher survival rate compared to WT YTHDF1 by enhancing antigen specific CD8+ T cell antitumor response. With depletion of YTHDF1 in dendritic cells, increased cross-presentation of tumor antigens and the cross-priming of CD8+ T cells was observed *in vivo*. It was found that lysosomal protease enzyme in dendritic cells with m6A was recognized by YTHDF1 (54).

After landmark discovery of PD1/PD-L1 and its role in immune evasion of cancer cells, the immune check point inhibitors (PD-1 inhibitors) have been established as potential anti-tumor agents. Immunotherapy has contributed immensely in terms of survival and quality of life in addition to chemoradiotherapy. The m6A modifications are also reported to play a key role in acquiring therapy resistance against checkpoint inhibitors. FTO inhibition suppresses melanoma tumorigenicity and increased the m6A methylation in PD-1, CXCR1, and SOX10 mRNA, henceforth enhancing the decay of mRNA by YTHDF2. Selective blocking of FTO restores IFN-γ response and sensitizes anti-PD-1 treatment *in vivo* (55). A study by Yi L et al. (56) suggest that m6A regulators are upregulated in HNSCC as compared to normal counterpart. Further they have demonstrated that m6A regulators show positive correlation with PDL-1 in tumor immune microenvironment (TIME), hence presenting the m6A regulators as viable therapeutic targets in HNSCC (56). Zhang et al. (57) suggested that low m6A scores activate immune cells to infiltrate TIME and increases the survival rate of gastric cancer patient compared with high m6A score with low survival rate. Low m6A score increased the neoantigen load as well as sensitized anti PDL-1 immunotherapy. Eraser protein ALKBH5 modulates TIME, deletion of ALKBH5 in colon and melanoma syngeneic tumor model enhances the immune cells infiltration in TIME. Mechanistically ALKBH5 modulates Mct4/Slc16a3 expression and lactate content in TIME, which ultimately suppressed the Treg and myeloid derived Cell. Deletion of ALKBH5 sensitizes the tumor against the anti PD-1 treatment and GVAX vaccine (58). Overall, these studies suggest that m6A methylation is a major regulator of immune response in tumor cells and TIME.

## Implication in Radiotherapy

Other than chemotherapy, radiation therapy is a major treatment regimen for cancer patients that target cancer cells by damaging the DNA. The concurrent chemo radio therapy is the most common therapeutic regimen followed by surgery (59). Radiation in GBM (glioblastoma) cells enhances the METTL3 expression and it increases the stability of SOX2 by recruiting hUR (human antigen R) and induces resistance against radiation (60). Similarly, selective knock down of METTL3 results in sensitizing pancreatic cancer lines to radiotherapy (38). Eraser protein FTO also induces chemo radio resistance in cervical squamous carcinoma by demethylation of β-catenin mRNA, which stabilizes its expression (30).

## Conclusion

**Figure 1:**
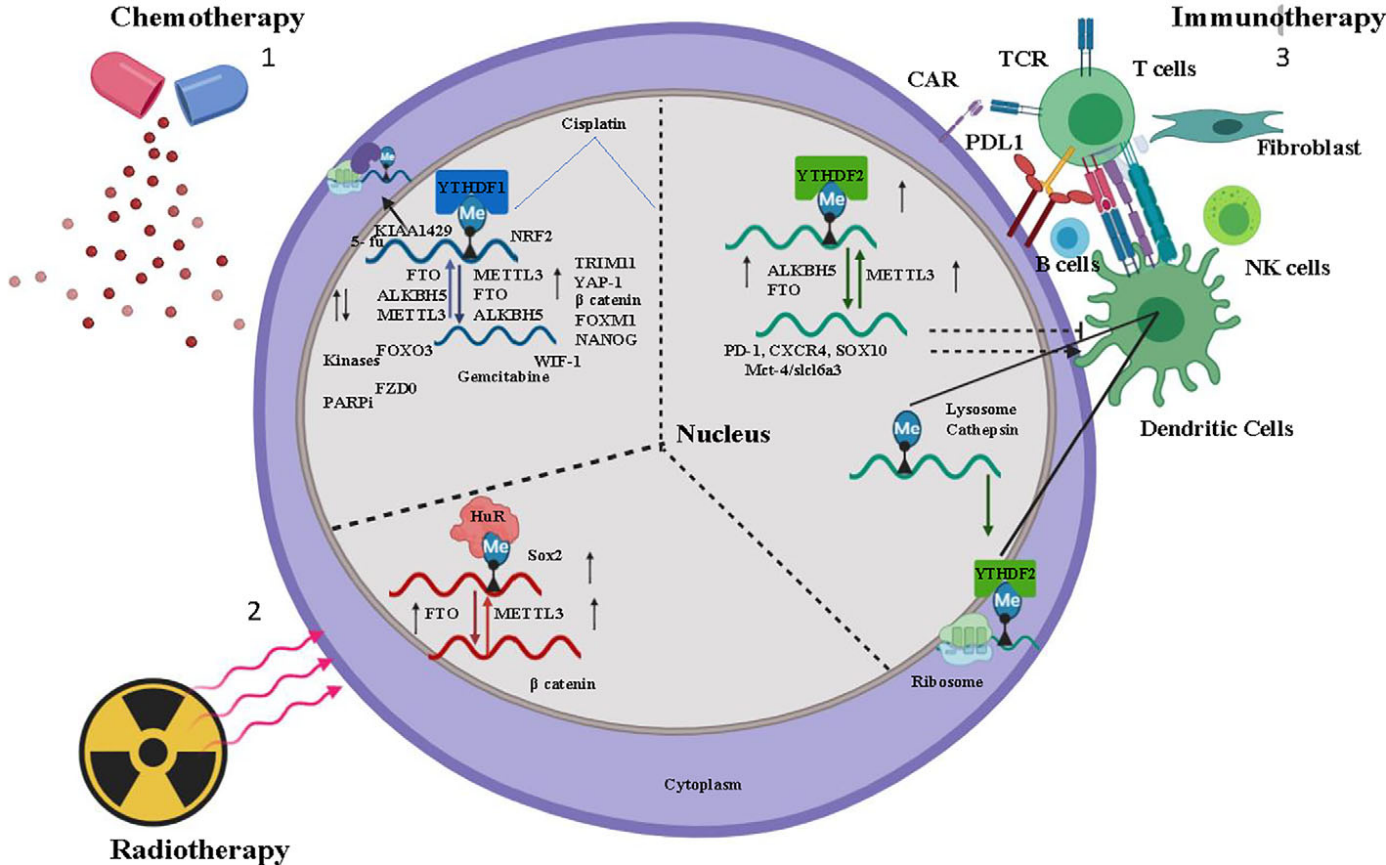
Overview of m6A regulation in Chemotherapy, Immunotherapy, and Radiotherapy- 1) Chemotherapeutic drugs Cisplatin, Kinase inhibitor modulates m6A regulator (Writer-METTL3, Raeder-YTHDF1, Eraser-ALKBH5, FTO) stabilizes the oncogene mRNA and induces chemoresistance. 2) Tumor immuno microenvironment (TIME) (B cells, dendritic cells, T cells, NK cells, fibroblast) activated by m6A regulator and induces immunotherapy resistance for cancer cells. 3) Radiation induces m6a regulator and stabilizes mRNA of cancer stem cell. In the figure, the small upward black arrow indicates “upregulation” and the downward black arrow indicates “down regulation”.

Therapy resistance in cancer is a consequence of multiple factors such as individual variability in sensitivity to the drug, location of the tumor, tissue lineage, tumor aggressiveness, and intracellular molecular alteration. As discussed earlier, deciphering the consequences of m6A modification on selective transcripts can lead to understanding the molecular mechanism of the therapy resistance (**Figure 1** and **Table 1**), thereby enabling us to optimize the combination therapy of existing drugs or to design specific drugs to overcome resistance property. However, the disadvantage lies on the insufficient studies regarding the selectivity of target mRNA by m6A methyltransferases, demethylases, and binding reader proteins. Along with that localization of m6A modified target transcripts, target specificity of m6A writer, reader, and eraser protein and their varied mode of action in different neoplasms remain unclear.

**Table 1:** Summary of m6A RNA modification in therapy resistant cancer cells.

Sr No.	Resistance	Cancer	m6A regulator	Onco/tumor suppressor	Ref
1	Cisplatin	CSCC	FTO	Oncogene	(30)
2	Cisplatin	Nasopharyngeal	METTL3	Oncogene	(28)
3	Cisplatin	NSCLC	METTL3	Oncogene	(19)
4	Cisplatin	NSCLC	YTHDF1	Tumor suppressor	(29)
5	Cisplatin	OSCC	ALKBH5	Oncogene	(31)
6	Cisplatin	Pancreatic cancer	METTL3	Oncogene	(38)
7	Cisplatin	NKTCL	WTAP	Oncogene	(61)
8	Cisplatin	Ovarian Cancer	YTHDF1	Oncogene	(62)
7	oxaliplatin	Colorectal cancer	YTHDF1	Oncogene	(39)
9	Doxorubicin	Osteosarcoma	m6A	Oncogene	(63)
10	Sorafenib	Hepatocellular carcinoma	METTL3	Tumor suppressor	(34)
11	Sorafenib	hepatocellular carcinoma	m6A	Oncogene	(64)
12	Afatinib	NSCLC	m6A	Oncogene	(33)
13	Crizotinib	NSCLC	METTL3/WTAP	Oncogene	(65)
14	Gefitinib	NSCLC	METTL3	Oncogene	(66)
15	5-Fu	Pancreatic Cancer	METTL3	Oncogene	(38)
16	5-Fu	Colorectal cancer	YTHDF1	Oncogene	(39)
17	Olaparib	Ovarian cancer	FTO/ALKBH5	Oncogene	(46)
18	Everolimus	Gastric cancer	METTL3	Tumor Suppressor	(67)
17	Tamoxifen	Breast Cancer	m6A	Oncogene	(68)
19	Y- Irradiation	GBM	METTL3	Oncogene	(60)
20	Y- Irradiation	Pancreatic cancer	METTL3	Oncogene	(38)
21	Y- Irradiation	CSCC	FTO	Oncogene	(30)
22	Anti PD1	Melanoma	FTO/YTHDF2	Oncogene	(55)
23	Anti PD1	Melanoma/colorectal	ALKBH5	Oncogene	(58)
24	PDL1	HNSCC	m6A	Oncogene	(56)

## Author Contributions

OS and RD designed the content of this review. OS, PM, and RD wrote the first draft of the manuscript. SM wrote sections of the manuscript. All authors contributed to the article and approved the submitted version.

## Funding

This work is supported by Institute of Life Sciences, Bhubaneswar intramural support, ICMR (5/13/9/2019-NCD-III) and SERB (CVD/2020/000154). OS is a UGC-SRF. PM is CSIR-JRF, and SM is UGC-JRF.

## Conflict of Interest

The authors declare that the research was conducted in the absence of any commercial or financial relationships that could be construed as a potential conflict of interest.
